# The differential impact of the DSM-5 post-traumatic stress symptoms on functional impairment in traumatized children and adolescents

**DOI:** 10.1007/s00787-023-02266-w

**Published:** 2023-08-02

**Authors:** Lasse Bartels, Ane-Marthe Solheim Skar, Marianne Skogbrott Birkeland, Silje Mørup Ormhaug, Lucy Berliner, Tine K. Jensen

**Affiliations:** 1 Department of Psychosomatics and Psychiatry, and Children’s Research Center, University Children’s Hospital Zurich, Steinwiesstrasse 75, 8032 Zurich, Switzerland; 2 Division of Child and Adolescent Health Psychology, Department of Psychology, University of Zurich, Zurich, Switzerland; 3 Norwegian Centre for Violence and Traumatic Stress Studies, Gullhaugveien 1-3, Nydalen, Oslo, Norway; 4 Harborview Abuse and Trauma Center, Patricia Steel Building, 401 Broadway, Suite 2075, Seattle, WA 98104 USA; 5 Department of Psychology, University of Oslo, Forskningsveien 3A, 0373 Oslo, Norway

**Keywords:** Children and adolescents, Functional impairment, Post-traumatic stress symptoms, Trauma

## Abstract

**Background:**

This study aims to provide a better understanding of the individual impact of the Diagnostic and Statistical Manual of Mental Disorders, Fifth Edition (DSM-5) post-traumatic stress symptoms (PTSS) on functional impairment in trauma-exposed children and adolescents. Identifying PTSS that have the most impact on functional impairment can broaden our understanding of post-trauma reactions and guide the selection of treatment components and techniques required to help patients to restore functioning following trauma exposure.

**Method:**

Utilizing relative importance analyses, unique shared variance of each DSM-5 PTSS with functional impairment were estimated in clinical samples of 3400 Norwegian (*M*_age_ = 14.18, *SD*_age_ = 2.49, range_age_ = 7–17) and 747 US (*M*_age_ = 10.76, *SD*_age_ = 3.10, range_age_ = 7–17) children and adolescents from naturalistic settings.

**Results:**

Negative beliefs, detachment from others, inability to experience positive emotions, and diminished interest in activities within the symptom cluster negative alterations in cognitions and mood, and the hyperarousal symptom concentration problems accounted for the largest proportions of unique variance explained in functional impairment in both samples. Further, the hyperarousal symptom irritability showed a unique high association with functional impairment in the US sample.

**Conclusion:**

As negative beliefs, emotional numbing symptoms, concentration problems and irritability may be especially related to functional impairment in traumatized children and adolescents, monitoring and targeting these symptoms throughout therapy might be of particular importance to restore functioning as early as possible and to facilitate overall recovery.

**Supplementary Information:**

The online version contains supplementary material available at 10.1007/s00787-023-02266-w.

## Introduction

Population-based and clinical-based studies indicate that a vast number of children and adolescents are exposed to one or several potentially traumatic events (PTEs) [22, 32]. Nearly 16% of children and adolescents develop post-traumatic stress disorder (PTSD) following trauma exposure [2]. PTSD is a highly heterogeneous disorder comprising internalizing and externalizing symptoms divided into clusters of intrusion, avoidance, negative cognition and mood, and hyperarousal [4]. PTSD often has an impact on children’s and adolescents’ social or educational spheres [21], compromising the ability to perform and take part in important and desired daily aspects of life.

Research endeavors, albeit only in adult samples, have shown a strong link between PTSD symptomatology and functional impairment(s) [14]. Given the highly differential nature of symptoms within the diagnostic criteria however, it is highly unlikely that all symptoms have an equal impairing impact [34]. Yet, thorough investigations in children and adolescents are missing. Whether certain symptoms might be more impairing than other is highly relevant from both a theoretical and a clinical perspective because patients exhibit large differences in symptom presentation. Furthermore, there is evidence that not all symptoms resolve at the end of treatment [20] and that symptom-related functional impairment may follow a chronic trajectory [30, 34]. Therefore, knowing the functionally most impairing PTSD symptoms in trauma-exposed children and adolescents would inform clinicians about the complex interplay between PTSS and symptom-related impairment in daily function. This might help tailor therapy according to the needs of patients from the start, and as a result, could help to prevent children and adolescents from following a negative developmental trajectory.

To date, only two studies of children and adolescents explored the associations between PTSD symptoms and functional impairment. Vibhakar [35] investigated the relationship between distinct PTSD symptoms and overall functional impairment in a sample of non-mental health treatment referred pre-adolescents and adolescents following an accident or unintentional injury. In the pre-adolescent group (7–11 years), the odds for functional impairment were increased in children exhibiting psychological cue reactivity, physiological cue reactivity, avoidance of thoughts/feelings, irritability, concentration problems, or hypervigilance. In the adolescent group (12–14 years), the odds for functional impairment was increased in adolescents exhibiting diminished interest in activities, irritability, concentration problems, or sleep problems. In a sample of 59 children and adolescents (7–14 years) exposed to interpersonal trauma, Carrion et al. [7] found significant relationships with overall functional impairment for the following PTSD symptoms: intrusive thoughts or memories, distressing dreams, flashbacks, trauma-related amnesia, diminished interest in activities, detachment from others, restricted affect, foreshortened future, concentration problems, and hypervigilance. It is important to note, that the low sample size might limit the generalizability of the findings, highlighting the need for further studies in large clinical samples of children and adolescents.

Summarized, the above-mentioned studies on symptom—impairment associations are based on the now-obsolete DSM-IV [3] PTSD criteria and mainly included samples of non-interpersonal trauma. Therefore, the aim of the current study was to extend the previous reports by identifying those PTSS with the greatest impact on functional impairment in a diverse clinical sample of trauma-exposed children and adolescents based on the current DSM-5 diagnostic algorithm. Research indicates that some of the new DSM-5 symptoms (i.e., negative beliefs and persistent negative emotional state) in the cluster negative alterations in cognitions and mood (NACM) might be central for the emergence and persistence of PTSD symptomatology in children and adolescence [5]. It may be particularly relevant to examine the association between these new criteria and functional impairment.

To the best of our knowledge, no study has yet investigated the unique associations between the DSM-5 defined PTSS and functional impairment in trauma-exposed children and adolescents. Therefore, we (a) test whether the DSM-5 PTSS have a differential impact on functional impairment and (b) examine the magnitude of differential DSM-5 PTSS impact on functional impairment. We expect that the individual DSM-5 PTSS differ in their impact on functional impairment. We followed an exploratory approach examining the differential magnitude of the impact of DSM-5 PTSS on functional impairment.

## Method

### Sample and study design

#### Norwegian sample

One data set was derived from a national implementation project with the goal of implementing trauma-focused Cognitive Behavioral Therapy (TF-CBT) in child and adolescent mental health services in Norway. Data on trauma-exposure, PTSS and functional impairment were collected as part of routine trauma and PTSD screening procedures at generalist regular child and adolescent mental health clinics at intake. The therapists were encouraged to assess PTEs and PTSS in the absence of the caregivers to facilitate unbiased responses. Further, the therapists were encouraged to be present when adolescents preferred to fill out the questionnaire independently in order to be able to answer any questions related to the items or to provide support when needed. All screening forms, also the self-completed, were followed by a clinical assessment by the therapist. No information on comorbidity was available, which may or may not have been screened for at the clinics. The data in the current study was collected from January 1, 2015, to December 31, 2017. Ethical approval was obtained by the Norwegian Regional Committee for Medical and Health Research (ref. 2017/1619 A). As the data was collected as part of routine screening and could not be associated with the individual child, the study received exemption to informed consent requirement.

Children and adolescents aged 6 and 18 years were included in the implementation project. However, only children and adolescents age 7–17 were included in the current study for comparability of the two samples. The other inclusion criteria were: (1) exposure to at least one PTE, (2) no missing values among the PTSS items (the R-packages used only apply listwise deletion), and (3) no missing values on all functional impairment items. See supplementary material (SM) 1 for information on the procedure of exclusion. Out of 23,506 children and adolescents, 3400 (*M*_age_ = 14.18, *SD*_age_ = 2.49, range_age_ = 7–17, female = 65.5%, male = 33.5%, no information on sex = 0.9%) fulfilled the inclusion criteria in the Norwegian sample. Information on ethnic background or other demographic variables was not available.

#### US sample

The US sample comprised retrospectively collected data of 824 clinical patients assessed at the Harborview Abuse & Trauma Center (Seattle, Washington, US), a clinic specialized in the treatment of child maltreatment, sexual assault, crime, and other traumas. Data on trauma exposure, PTSS, and functional impairment were collected as part of routine trauma and PTSD screening procedures at the center at intake. No information on comorbidity was available, which may or may not have been screened for at the center. Children and adolescents were routinely screened (≤ 11 years: interview; ≥ 12 years: self-report) for trauma exposure and PTSS at intake in the absence of their caregivers. The therapist was always present when children and adolescents filled out the questionnaire on their own in case of difficulties in understanding. Further, information about age, gender, race, and ethnicity was collected. Data in the current study was collected from August 14, 2014 to March 12, 2020. The IRB at site allowed the use of previously collected, de-identified data, based on the ruling that it does not constitute human research subjects. Therefore, a full IRB review was not required. Inclusion criteria were the same as for the Norwegian sample. Subsequently, the US sample comprised 747 children and adolescents (*M*_age_ = 10.76, *SD*_age_ = 3.10, range_age_ = 7–17, female = 65.1%, male = 34.7%, transgender = 0.3%). See SM1 for information on the exclusion procedure. Children and adolescents racial background included White (50.3%), African American (15.0%), other racial background (13.4%), Multiracial (10.6%), Asian (4.6%), Pacific Islander (2.8%), and Native American (2.0%). Information on racial background was not reported for 10 (1.3%) children and adolescents. Overall, 150 (20.1%) children and adolescents were Hispanic.

### Measures

#### Potentially traumatic events

To assess PTEs, the Child and Adolescents Trauma Screen (CATS; [29], comprising 15 items, was used. In the Norwegian version of the questionnaire, the questions were slightly different, with bullying as an additional exposure item (See Table 1).

**Table 1:** Overview of potentially traumatic events

	Norway		US	
	*N*^*a*^	*n*/%	*N*^*a*^	n/%
Accidents	3351	666/19.6	747	167/22.4
Natural disasters	3348	437/12.9	747	39/5.2
War/terror^d^	3371	149/4.4	747	6/0.8
Witnessing community violence^d^	3339	979/28.8	746	135/18.1
Experiencing community violence^d^	3349	1029/30.3	747	122/16.3
Witnessing family violence^d^	3340	860/25.3	747	255/34.1
Experiencing family violence^d^	3338	1046/30.8	743	200/26.8
Sexual abuse^d^	3381	898/26.6	747	489/65.5
Medical	3338	966/28.4	747	157/21.0
Other traumatic event	3081	1192/35.1	746	335/44.8
Severe illness/sudden death of close person^b^	3323	1996/58.7		
Bullying with threats^b,d^	3314	1855/54.6		
Kidnapping^b,d^	3354	217/6.4		
Serious illness or sudden or violent death of close person^c^			747	206/27.6
Robbed by threat, force, or weapon^c,d^			747	59/7.9
Attacked, stabbed, shot at, hurt badly^c,d^			747	69/9.2
Witnessing attack, stabbing, shooting, killing^c,d^			747	96/12.9

^a^Total number of responses in the samples, which *n*/% are based on

^b^Only Norwegian version

^c^Only US version

^d^Interpersonal traumatic events

#### Post-traumatic stress symptoms

The frequency of the 20 DSM-5 PTSS within the last two weeks in both samples were assessed via the CATS on a 4-point Likert scale (0 =  “Never”, 1 =  “Once in a while”, 2 =  “Half of the time” and 3 =  “Almost always”). The total symptom severity score ranges from 0 to 60. Psychometric validation analyses showed high internal consistency with Cronbach’s *α* = 0.92 [29]. Cronbach’s *α* was 0.93 in the Norwegian sample and 0.94 in the US sample.

#### Functional impairment

The CATS measures impairment in psychosocial functioning dichotomously (Yes/No) across five domains: (1) getting along with others, (2) hobbies/fun, (3) school or work, (4) family relationships, and (5) general happiness. The five functional impairment domain items were summed to obtain a measure of overall impairment.

### Statistical analyses

Two analysis steps were performed, for which the statistical procedure of Fried and Nesse [9] were followed.

#### Varying impact of PTSS on overall impairment

First, two linear regression analyses were conducted using the 20 DSM-5 PTSS to predict overall functional impairment via Mplus v8.3 [24]. Maximum likelihood parameter estimators with robust standard errors and a mean- and variance-adjusted χ^2^ test (i.e., MLMV) were used. We opted not to control for covariates associated with PTSD symptomatology (e.g., sex/gender, number, and type of trauma exposure) to avoid loss of variance due to over adjusting for processes that are on the pathway from the trauma exposure to PTSS to impairment. In the first linear regression model (heterogeneity model), regression weights for the symptoms were free to vary. The assumption was that PTSS were allowed to have differential associations with overall functional impairment. In model II (homogeneity model), the regression weights were constrained to be equal. Here, the assumption was that all symptoms are equally associated with overall functional impairment. A χ^2^ test was conducted to compare model fit. Further, we conducted multicollinearity diagnostics because of the general assumption of associations among the 20 DSM-5 PTSS.

#### Relative importance of PTSS

Second, we conducted relative importance analysis via R (v4.0.2; [27]) to examine the magnitude of impact on functional impairment for each of the 20 DSM-5 PTSS. Compared to commonly used measures of relative importance such as bivariate correlations and standardized regression coefficients, relative importance metrics reflect the unique contribution each predictor makes to the total explained variance (*R*^2^) in the criterion by itself and its contribution when combined with other predictors [16]. Thus, relative importance metrics allow us to draw more accurate inferences concerning each predictor’s relative contribution to the criterion in case of correlated predictors. To estimate the relative importance (i.e., unique proportion of explained variance) of each of the 20 DSM-5 PTSS, the *lmg* metric from the R-package relaimpo [10] was used. The metric lmg decomposes the total explained *R*^2^ of the model into one non-negative proportion of explained variance for each predictor. In the process of decomposing, the relative importance of each predictor is obtained by averaging the proportions of variances calculated for each predictor at all possible entry points in the model. The relative importance estimates sum to the total explained *R*^2^ of the model. Further, we rescaled the relative importance estimates to sum to 100%, representing the percentage of the total explained *R*^2^ of the model associated with each predictor. Further, confidence intervals (CIs) for the rescaled relative importance estimates were calculated using the implemented bootstrap procedure. Additionally, we tested whether the 20 DSM-5 PTSS differed significantly in their relative importance contribution, i.e., whether symptom A contributes to explain significantly more or less of impairment than symptom B does (thus A and B is a pair) across all 190 possible symptom pairs ((20*19)/2). Following Fried and Nesse [9], we further tested if differences in PTSS impact on impairment were due to the symptom nature or due to their severity. If symptom severity is causing large differences between the PTSS, then a large proportion of the differences of the RI estimates should be explained by differences in symptom severity (i.e. PTSS with a high mean symptom severity have a greater impact on impairment than PTSS with a low mean symptom severity). We tested this hypothesis using a linear regression, predicting the relative importance estimate of each of the 20 PTSS by its mean symptom severity. Data were assumed to be interval level.

## Results

In the Norwegian sample, 3.21 (*SD*_PTE_ = 2.22) PTEs were experienced on average, and 2865 (84.3%) children and adolescents experienced more than one PTE. Further, 2862 (84.2%) experienced some type of interpersonal trauma, and 72 (2.1%) children and adolescents only reported other traumatic events. In the US sample, 3.45 (*SD*_PTE_ = 2.22) PTEs were experienced on average. A total of 606 (81.1%) children and adolescents experienced more than one PTE. Moreover, 671 (89.8%) children and adolescents experienced some type of interpersonal trauma, and 32 (4.3%) children and adolescents only reported other traumatic event. The average CATS sum score was *M* = 21.93 (*SD* = 13.81) for the Norwegian sample and *M* = 26.24 (*SD* = 14.21) for the US sample. On average, the Norwegian sample experienced functional impairment in 2.44 (*SD*_impairment_ = 1.82) life domains and the US sample in 2.99 life domains (*SD*_impairment_ = 1.79). See SM2 for mean and standard deviation of each PTSS.

### Varying impact of PTSS on overall impairment

#### Norwegian sample

The fit of the heterogeneity model was significantly better than the homogeneity model (*χ*^*2*^ = 309.36, *df* = 19, *p* < 0.001). Among the 20 PTSS, 13 symptoms predicted significantly overall impairment (see SM3), explaining 55.03% of the variance [*F*(20, 3379) = 206.7, *p* < 0.001]. Additionally, multicollinearity diagnostics indicated no multicollinearity problems as the variance inflation factors did not exceed 2.5 for any PTSS (see SM3) [17].

#### US sample

The heterogeneity model fitted the data significantly better than the homogeneity model (*χ*^*2*^ = 120.41, *df* = 19, *p* < 0.001). Out of the 20 PTSS, 9 symptoms predicted significantly overall impairment (see SM3), explaining 43.4% of the variance [*F*(20, 726) = 27.78, *p* < 0.001]. Additionally, multicollinearity diagnostics indicated no multicollinearity problems as the variance inflation factors did not exceed 2.5 for any PTSS (see SM3) [17].

### Relative importance of PTSS

#### Norwegian sample

**Fig. 1:**
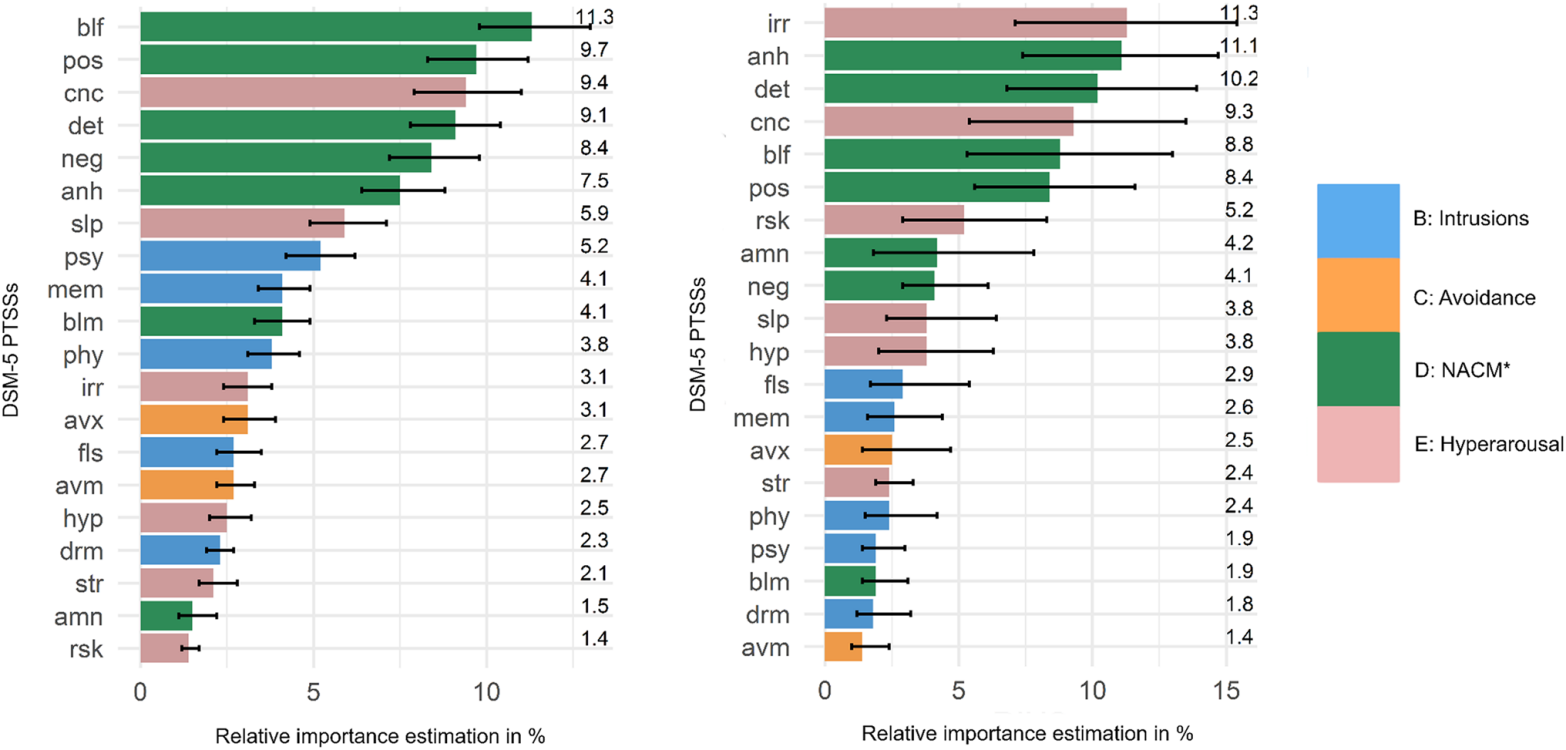
Relative importance estimates of the DSM-5 PTSS on impairment in the Norwegian (left) and US (right) sample, including bootstrapped 95% confidence intervals. Values (adjusted to sum to 100%) represent the unique *R*^2^ contribution of a PTSS on impairment. *NACM = negative alterations in cognitions and mood; mem = intrusive thoughts or memories, drm = distressing dreams, fls = dissociative flashbacks, psy = psychological cue reactivity, phy = physiological cue reactivity, avm = avoidance of thoughts or memories, avx = avoidance of external reminders, amn = trauma-related amnesia, blf = negative beliefs, blm = distorted blaming of oneself or others, neg = persistent negative emotional state, anh = diminished interest in activities, det = detachment from others, pos = inability to experience positive emotions, irr = irritability, rsk = self-destructive or reckless behavior, hyp = hypervigilance, str = exaggerated startle response, cnc = concentration problems, and slp = sleep disturbance

The 20 DSM-5 PTSS contributed to the total explained variance in a range from 1.4% (self-destructive or reckless behavior) to 11.3% (negative beliefs) (Fig. 1). The PTSS negative beliefs (11.3%), inability to experience positive emotions (9.7%), concentration problems (9.4%), detachment from others (9.1%), persistent negative emotional state (8.4%), and diminished interest in activities (7.5%) accounted for the largest proportions of unique variance explained in overall impairment (Fig. 1). Out of 190 PTSS pairs, 142 (74.74%) differed significantly in their relative importance contribution to overall impairment (all *p* < 0.05) (SM4a). Further, the results [*F*(1, 18) = 4.935, *p* = 0.04] indicate that the differences in relative importance estimates were potentially due to the mean symptom severity rather than the unique nature of the PTSS.

#### US sample

The 20 DSM-5 PTSS contributed to the total explained variance in a range from 1.4% (avoidance of intrusive thoughts or memories) to 11.3% (irritability) (Fig. 1). The PTSS irritability (11.3%), diminished interest in activities (11.1%), detachment from others (10.2%), concentration problems (9.3%), negative beliefs (8.8%), and inability to experience positive emotions (8.4%) accounted for the largest proportions of unique variance explained in functional impairment. Out of 190 PTSS pairs, 92 (48.42%) differed significantly in their relative importance contribution to impairment (all *p* < 0.05) (SM4b). As for the Norwegian sample, we tested if the large differences in PTSS impact on impairment were due to the symptom nature or due to their severity. The non-significant result [*F*(1, 18) = 0.005, *p* = 0.94] indicates that the differences in relative importance estimates were due to the unique nature of the PTSS and not due to the mean symptom severity.

## Discussion

This is the first study to explore the unique impact of the DSM-5 PTSS on functional impairment in a clinical trauma-exposed child and adolescent sample utilizing relative importance analysis. As expected, individual PTSS exhibited differential impact on functional impairment, both in the US and the Norwegian sample. Our results indicate that symptoms in the NACM and hyperarousal clusters explained the largest proportions of variance in functional impairment. The PTSS of re-experiencing and active avoidance played a less influential role. The NACM cluster contains many non-specific symptoms that have significant overlap with common symptoms of depression and anxiety. Emotional numbing symptoms (i.e. diminished interest in activities, detachment from others, and inability to experience positive emotions) are very similar to withdrawal and anhedonia characteristic of depression. The NACM cluster has two symptoms related to cognitions; one that reflects core beliefs (negative beliefs about self or others) and another that is trauma-specific (self or other blame for what happened). The more generalized negative cognition in the present study has more influence on impairment. Negative beliefs are also symptoms of depression. The hyperarousal cluster contains common transdiagnostic symptoms as well as some that are highly specific to PTSD (e.g., hypervigilance, exaggerated startle). The hyperarousal symptoms with the strongest relationships with impairment in the present study are common in depression and anxiety (e.g., sleep difficulties, concentration problems, irritability). Overall, these findings suggest that the PTSS that are not trauma-specific have the most impact on impairment.

The most consistent finding from the present study and Vibhakar [35] and Carrion et al. [7] is the strong association between diminished interest in activities and concentration problems and overall functional impairment. Withdrawal, whether PTSD- or depression-related, may lead to a number of social consequences such as impaired communication, reduced opportunities to experience positive emotions [11], decreased participation in activities of importance in peer relationships, school attendance, and isolation. This could in turn hinder the child or adolescent’s peer relationships from interacting in ways that nurture or sustain them. Concentration problems might be especially disruptive when children and adolescents are engaged in daily or meaningful activities. Indeed, studies have found that children and adolescents perform worse academically following trauma exposure (e.g., [33]). Further, the strong association between concentration problems and functioning is consistent with findings in the adult literature [9, 18]. Moreover, and consistent with Vibhakar [35], irritability showed also a strong link to overall functional impairment in the present study’s US sample. This association has also been found in depressed children and adolescents [31]. Thus, irritability might be a transdiagnostic risk factor for functional impairment throughout childhood and especially warrants clinical attention. Interestingly, irritability made a larger contribution to functional impairment and was more frequently exhibited in the US sample than in the Norwegian sample. Since studies on physical aggression among children and adolescents seem to find higher rates in the US [19] than in Norway [12], this finding might mirror cultural differences. Consistent with Carrion et al. [7], detachment from others and the inability to experience positive emotions appear to have strong influence on functional impairment. These findings are also in accordance with the adult literature (e.g., [28]). It seems reasonable that these symptoms can interfere with relationships, as individuals exhibiting these symptoms may be reluctant to engage in activities, they previously enjoyed [28]. In contrast to the results of the present study, Carrion et al. [7] and Vibhakar [35] found that re-experiencing symptoms, avoidance, and hyperarousal beyond concentration problems predict impairment. Avoidance might be specific to accidental trauma, whereas dysfunctional cognitions and negative emotions may play a more salient role in children and adolescents exposed to interpersonal trauma [6]. Since a large proportion of our samples (Norway = 83.3%, US = 89.8%) experienced interpersonal trauma compared to Vibhakar [35], emotional numbing and dysfunctional cognitions rather than avoidance may have played a larger functional impairing role. Future research needs to replicate these findings across samples controlling for possible confounding variables, such as type of PTE. It is important to note that the comparability of the results between the present study and Carrion et al. [7] might be limited due to the different statistical approaches taken. Carrion et al. [7] conducted simple linear regressions (i.e., a separate model for each symptom). This approach bears the risk of misleading associations due to excluding confounding predictors [26], which in this case would be the other PTSS. Replication studies are needed to compare findings of differential statistic approaches directly.

There is a strong link between negative cognitions and the onset and maintenance of PTSD as well as PTSS severity in children and adolescents [23]. Our findings demonstrate that overall functional impairment is affected by distorted appraisals. A traumatic experience can deeply disrupt a child or adolescent's trust in others and undermine a positive view of self, as the trauma may have been caused by family members and/ or peers that were supposed to be trustworthy. Consequently, it may be that children and adolescents withdraw from others to avoid future traumatic experiences. Further, perceiving the world as dangerous may lead to an increased expectation of outcomes in future neutral events. Thus, children and adolescents may stop participating in activities that are of significance to their peer relationships and education. It is curious that the cardinal PTSD symptoms (re-experiencing, avoidance, and hypervigilance/startle) do not show a strong association with impairment in our samples. Clinically, these symptoms are highly distressing to patients.

In addition to diagnosis and the PTSS clinical intensity score, an expanded assessment of individual symptoms and their relationship to functional impairment should be incorporated into clinical case conceptualization. A process-based therapy approach [13] could aid clinicians to engage in a more fine-grained, individualized assessment of functional impairments in the initial stage of therapy. More specifically, clinicians should consider the functions of the child’s thoughts, behaviors, and emotions (including symptoms of comorbid disorders states) and their relationships with various domains of functioning jointly defined with the child or adolescent. Doing so, patients ought to provide a narrative description of functional impairments besides the assessment via standardized instruments [28]. The present study suggests that negative beliefs, detachment from others, inability to experience positive emotions, diminished interest in activities, concentration problems and irritability mainly drive functional impairment in children and adolescents. However, it remains unclear to what extent other symptoms influence these symptoms, how symptoms interact over time, and how dynamic interrelations impact functional impairment. Thus, a clinically pragmatic implication based on the cross-sectional nature of the present study is that clinicians should routinely apply functional analysis jointly with the patient on a session-by-session basis. In doing so, clinicians would be able to “plot” out the net of domains of functioning the child or adolescent might be struggling with and to connect these to specific behaviors, cognitions, and emotions. This would aid clinicians to decide whether to modify or add specific interventions throughout the therapy. A strength of this study was the use of two large, diverse clinical samples. The fact that the findings are similar may point to something generic in PTSD and functioning, at least in western societies. Further, a relative importance approach was applied to the current PTSD conceptualization (DSM-5), allowing for correct partitioning of predicted variance to the appropriate PTSS. The results of this study should be interpreted in light of several limitations. PTSS and functional impairment were assessed at the same time point. Since PTSS and functional impairment might follow a bidirectional path [15], causality has to be interpreted with caution and longitudinal designs are needed to provide insights into causal dynamics between PTSS and functioning. However, it is important to note that the instruments used in this study specifically asked children and adolescents if and how the present PTSS were interfering with their functioning and not the other way around. Further, we could not control for comorbidity. Further, the bootstrapped confidence intervals for the relative importance estimates in the US sample were fairly large, given the smaller sample size. Thus, a moderate amount of model uncertainty due to the large number of predictors was implied. Moreover, differential variability in PTSS potentially drove the magnitude of relative importance in the Norwegian but not in the US sample, since symptom means significantly predicted the estimates. However, even the PTSS with the lowest and highest mean in the Norwegian study demonstrated considerable variability (see SM2). Therefore, statistical significance could have been reached due to the large sample size and statistical power rather than due to skewness of PTSS. More studies are needed to gain a better understanding of symptom variability and its impact on functional impairment. Working within the possibilities of the R package, 540 of 4344 cases were excluded from the Norwegian sample due to missing data on the item level. However, symptom levels in this group were largely not significantly different from symptom levels in the group with a complete dataset (see SM5). Still, there is a possibility that this could have potentially introduced bias in results. Lastly, overall functional impairment was the sum of five binary responses to impairments in functioning. Thus, a child or adolescent with mild impairments in several domains could have been treated as more impaired than a child or adolescent with severe impairment in two areas. It is therefore important for future replication studies to include a continuous scale. It is important to note, that a symptom-based approach as used in the present study does not differentiate between ‘PTSD-related concentration problems’ and ‘depression-related concentration problems’ in trauma-exposed individuals. Future studies should therefore examine symptoms that do not overlap with the DSM-5 PTSD criteria to inform our understanding of functional impairment as children and adolescents exposed to PTEs often present with comorbid depression and anxiety disorders (e.g., [1, 8]). Further, longitudinal studies with within-subjects designs will be needed to explore the temporal dynamics between distinct trauma-related symptoms (i.e., PTSD symptomatology and comorbid symptoms) and functional impairment(s). Utilizing experience sampling methodology (ESM; [25]) in the form of digital diaries could inform researchers about the development of such symptom-related burdens in children and adolescents. Future studies should also include clinical interviews as a complement to self-report measures in evaluating symptom-impairment relationships.

Altogether, our findings add clinically relevant information about the impact of the DSM-5 PTSS on functional impairment in children and adolescents. The most impairing PTSS were negative beliefs, loss of interest, detachment from others, inability to experience positive emotions, and concentration problems. Monitoring and targeting these symptoms throughout therapy to restore functioning as early as possible may be crucial for recovery for traumatized children.

### Supplementary Information

Below is the link to the electronic supplementary material.

Supplementary file1 (DOCX 50 KB)

## Data Availability

Given that the ethical committees agree, data from the present study will be available on request from the principle investigator.

## References

[1] Adams ZW, Danielson CK, Sumner JA, McCauley JL, Cohen JR, Ruggiero KJ (2015). Comorbidity of PTSD, major depression, and substance use disorder among adolescent victims of the spring 2011 tornadoes in alabama and joplin. Missouri Psychiatry.

[2] Alisic E, Zalta AK, Van Wesel F, Larsen SE, Hafstad GS, Hassanpour K, Smid GE (2014). Rates of post-traumatic stress disorder in trauma-exposed children and adolescents: meta-analysis. Br J Psychiatry.

[3] American Psychiatric Association (1994). Diagnostic and statistical manual of mental disorders.

[4] American Psychiatry Association (2013). Diagnostic and statistical manual of mental disorders.

[5] Bartels L, Berliner L, Holt T, Jensen T, Jungbluth N, Plener P, Sachser C (2019). The importance of the DSM-5 posttraumatic stress disorder symptoms of cognitions and mood in traumatized children and adolescents: two network approaches. J Child Psychol Psychiatry Allied Discip.

[6] Birkeland MS, Skar AMS, Jensen TK (2022). Understanding the relationships between trauma type and individual posttraumatic stress symptoms: a cross-sectional study of a clinical sample of children and adolescents. J Child Psychol Psychiatry.

[7] Carrion VG, Weems CF, Ray R, Reiss AL (2002). Toward an empirical definition of pediatric PTSD: the phenomenology of PTSD symptoms in youth. J Am Acad Child Adolesc Psychiatry.

[8] Copeland WE, Keeler G, Angold A, Costello EJ (2007). Traumatic events and posttraumatic stress in childhood. Arch Gen Psychiatry.

[9] Fried EI, Nesse RM (2014). The impact of individual depressive symptoms on impairment of psychosocial functioning. PLoS One.

[10] Grömping U (2006). Relative importance for linear regression in R: the package relaimpo. J Stat Softw.

[11] Hassija C, Cloitre M (2015). STAIR narrative therapy: a skills focused approach to trauma-related distress. Curr Psychiatry Rev.

[12] Henriksen M, Skrove M, Hoftun GB, Sund ER, Lydersen S, Tseng WL, Sukhodolsky DG (2021). Developmental course and risk factors of physical aggression in late adolescence. Child Psychiatry Hum Dev.

[13] Hofmann SG, Hayes SC (2019). The future of intervention science: process-based therapy. Clin Psychol Sci.

[14] Jellestad L, Vital NA, Malamud J, Taeymans J, Mueller-Pfeiffer C (2021). Functional impairment in posttraumatic stress disorder: a systematic review and meta-analysis. J Psychiatr Res.

[15] Johnson DM, Palmieri PA, Jackson AP, Hobfoll SE (2007). Emotional numbing weakens abused inner-city women’s resiliency resources. J Trauma Stress.

[16] Johnson JW, LeBreton JM (2004). History and use of relative importance indices in organizational research. Organ Res Methods.

[17] Johnston R, Jones K, Manley D (2018). Confounding and collinearity in regression analysis: a cautionary tale and an alternative procedure, illustrated by studies of British voting behaviour. Qual Quant.

[18] Kachadourian LK, Harpaz-Rotem I, Tsai J, Southwick SM, Pietrzak RH (2019). Posttraumatic stress disorder symptoms, functioning, and suicidal ideation in us military veterans: a symptomics approach. Prim Care Companion CNS Disord.

[19] Kann L, McManus T, Harris WA, Shanklin SL, Flint KH, Queen B, Ethier KA (2018). Youth risk behavior surveillance-United States 2017. MMWR Surveill Summ.

[20] Larsen SE, Fleming C, Resick PA (2019). Residual symptoms following empirically supported treatment for PTSD. Psychol Trauma Theory Res Pract Policy.

[21] Lewis SJ, Arseneault L, Caspi A, Fisher HL, Matthews T, Moffitt TE, Danese A (2019). The epidemiology of trauma and post-traumatic stress disorder in a representative cohort of young people in England and Wales. Lancet Psychiatry.

[22] McLaughlin KA, Koenen KC, Hill ED, Petukhova M, Sampson NA, Zaslavsky AM, Kessler RC (2013). Trauma exposure and posttraumatic stress disorder in a national sample of adolescents. J Am Acad Child Adolesc Psychiatry.

[23] Mitchell R, Brennan K, Curran D, Hanna D, Dyer KFW (2017). A meta-analysis of the association between appraisals of trauma and posttraumatic stress in children and adolescents. J Trauma Stress.

[24] Muthén BO, Muthén L (2012). Mplus user’s guide.

[25] Myin-Germeys I, Kasanova Z, Vaessen T, Vachon H, Kirtley O, Viechtbauer W, Reininghaus U (2018). Experience sampling methodology in mental health research: new insights and technical developments. World Psychiatry.

[26] PennState Eberly College of Science (n.d.) 10.10—other regression Pitfalls. Retrieved 8 Mar 2022. from https://online.stat.psu.edu/stat462/node/185/

[27] R CoreTeam (2014) R: a language and environment for statistical computing. R Foundation for Statistical Computing, Vienna, Austria

[28] Rodriguez P, Holowka DW, Marx BP (2012). Assessment of posttraumatic stress disorder-related functional impairment: a review. J Rehabil Res Dev.

[29] Sachser C, Berliner L, Holt T, Jensen TK, Jungbluth N, Risch E, Goldbeck L (2017). International development and psychometric properties of the Child and Adolescent Trauma Screen (CATS). J Affect Disord.

[30] Scheeringa MS, Zeanah CH, Myers L, Putnam FW (2005). Predictive validity in a prospective follow-up of PTSD in preschool children. J Am Acad Child Adolesc Psychiatry.

[31] Sherwood SN, Youngstrom JK, Findling RL, Youngstrom EA, Freeman AJ (2021). Irritability is associated with illness severity and anhedonia is associated with functional impairment among depressed children and adolescents. J Child Adolesc Psychopharmacol.

[32] Skar AMS, Ormhaug SM, Jensen TK (2019). Reported levels of upset in youth after routine trauma screening at mental health clinics. JAMA Netw Open.

[33] Strøm IF, Schultz JH, Wentzel-Larsen T, Dyb G (2016). School performance after experiencing trauma: a longitudinal study of school functioning in survivors of the Utøya shootings in 2011. Eur J Psychotraumatol.

[34] Tanner J, Zeffiro T, Wyss D, Perron N, Rufer M, Mueller-Pfeiffer C (2019). Psychiatric symptom profiles predict functional impairment. Front Psych.

[35] Vibhakar V (2018) The manifestation of post—traumatic stress in children and young persons aged 7–14 years and implication s for PTSD diagnosis. PhD Thesis, Faculty of Medicine, The University of Queensland, Queensland, Australia. 10.14264/uql.2018.853

