# Supplementary material for: The differential impact of the DSM-5 post-traumatic stress symptoms on functional impairment in traumatized children and adolescents
Source: Eur Child Adolesc Psychiatry. 2023 Aug 2;33(5):1573–81. doi: 10.1007/s00787-023-02266-w (PMC11098905; doi:10.1007/s00787-023-02266-w)
Supplement: Supplementary file 1 — Supplementary file1 (DOCX 50 KB) [file 787_2023_2266_MOESM1_ESM.docx]

**The differential impact of the DSM-5 post-traumatic stress symptoms on functional impairment in traumatized children and adolescents**

Supplementary materials

Bartels, [Skar](https://link.springer.com/article/10.1007/s00787-019-01340-6#auth-2), Birkeland, Ormhaug, Berliner, Jensen, 2023

E-mail address of the corresponding author: [lasse.bartels@kispi.uzh.ch](mailto:lasse.bartels@kispi.uzh.ch)

SM1. Exclusion process within samples

*Norwegian sample*

The overall Norwegian sample comprised 23506 cases from generalist regular child and adolescent mental health clinics

1. A total of 9924 cases were excluded because no information on age was available. Further, 296 children were excluded because they were younger than 7 years, and 153 children because they were older than 17 years.
2. Further, 2304 cases were excluded because no information on the trauma index was available and 2549 cases were excluded because they reported no trauma.
3. Next, 3936 cases who had reported at least event were not screened for PTSS (thus there are no symptom data), due to clinical decisions by the therapists (probably because the therapists considered that the event did not fulfill criterion A, or because there were other more pertinent mental health problems that were more relevant to screen for). Thus, 4344 were screened for PTSS.
4. Since the R-package used in the present study only includes cases with no missing values on the independent variables and dependent variable, further 540 cases were excluded because of on ore more missing values among the 20 DSM-5 PTSS and 403 cases were excluded because of complete missing values on the 5 functional impairment items. Further, one case was excluded because it scored a value of 3 on the functional impairment item “getting along with others”, which is not possible due to dichotomously nature of the item (Yes/No).

*US sample*

The overall US sample comprised 824 cases from a specialized trauma clinic.

1. Out of the 824 total cases, seven cases were excluded because no information on the trauma index was available and 58 cases were excluded because they reported no trauma.
2. Since the R-package used in the present study only includes cases with no missing values on the independent variables and dependent variable, 12 cases were excluded because of missing values among the 20 DSM-5 PTSS.

| SM2  Mean and standard deviation for each of the 20 DSM-5 PTSS | | | | |
| --- | --- | --- | --- | --- |
|  | Norwegian sample  (*n* = 3400) | | US sample  (*n* = 747) | |
|  | *M* | *SD* | *M* | *SD* |
| Intrusive thoughts or memories | 1.14 | 0.97 | 1.30 | 1.02 |
| Distressing dreams | 0.73 | 0.94 | 1.05 | 0.98 |
| Dissociative flashbacks | 0.67 | 0.90 | 0.75 | 0.92 |
| Psychological cue reactivity | 1.42 | 1.08 | 1.81 | 1.08 |
| Physiological cue reactivity | 1.15 | 1.09 | 1.25 | 1.12 |
| Avoidance of thoughts or memories | 1.62 | 1.15 | 1.86 | 1.11 |
| Avoidance of external reminders | 1.27 | 1.18 | 1.58 | 1.18 |
| Trauma-related amnesia | 0.83 | 1.04 | 0.96 | 1.05 |
| Negative beliefs | 1.35 | 1.17 | 1.58 | 1.15 |
| Distorted blaming of oneself or others | 0.95 | 1.11 | 1.39 | 1.15 |
| Persistent negative emotional state | 1.40 | 1.12 | 1.75 | 1.04 |
| Diminished interest in activities | 0.98 | 1.11 | 1.11 | 1.08 |
| Detachment from others | 1.05 | 1.12 | 1.30 | 1.07 |
| Inability to experience positive emotions | 0.96 | 0.99 | 1.19 | 0.99 |
| Irritability | 0.91 | 0.99 | 1.43 | 1.04 |
| Self-destructive or reckless behavior | 0.41 | 0.76 | 0.69 | 0.89 |
| Hypervigilance | 1.09 | 1.14 | 1.16 | 1.11 |
| Exaggerated startle response | 0.99 | 1.12 | 1.02 | 1.04 |
| Concentration problems | 1.62 | 1.17 | 1.61 | 1.05 |
| Sleep disturbance | 1.40 | 1.20 | 1.46 | 1.12 |
|  | | | | |

| SM3  Unstandardized regression coefficients, standard errors, p-values of the linear regression analysis, and variance inflation factors for each PTSS (heterogeneity models) | | | | | | | | |
| --- | --- | --- | --- | --- | --- | --- | --- | --- |
|  | Norwegian sample | | | | US sample | | | |
| DSM-5 posttraumatic stress symptoms | *b* | *s.e.* | *p* | *VIF* | *s.e.* | *p* | *b* | *VIF* |
| Intrusive thoughts or memories | .09 | .03 | .005 | 1.94 | .03 | 0.07 | .634 | 2.12 |
| Distressing dreams | -.03 | .03 | .288 | 1.68 | -.05 | 0.07 | .490 | 1.97 |
| Dissociative flashbacks | .06 | .03 | .039 | 1.51 | .11 | 0.07 | .149 | 1.83 |
| Psychological cue reactivity | .11 | .03 | .000 | 2.21 | -.08 | 0.07 | .287 | 2.48 |
| Physiological cue reactivity | .02 | .03 | .538 | 2.07 | .01 | 0.07 | .890 | 2.19 |
| Avoidance of thoughts or memories | -.02 | .02 | .524 | 1.69 | -.11 | 0.06 | .064 | 1.87 |
| Avoidance of external reminders | .06 | .02 | .005 | 1.57 | .07 | 0.06 | .223 | 1.95 |
| Trauma-related amnesia | .04 | .02 | .049 | 1.19 | .15 | 0.05 | .008 | 1.33 |
| Negative beliefs | .25 | .03 | .000 | 2.20 | .23 | 0.07 | .001 | 2.36 |
| Distorted blaming of oneself or others | .04 | .02 | .122 | 1.65 | -.06 | 0.06 | .299 | 1.82 |
| Persistent negative emotional state | .16 | .03 | .000 | 2.30 | -.06 | 0.07 | .408 | 2.34 |
| Diminished interest in activities | .16 | .03 | .000 | 1.80 | .26 | 0.07 | .000 | 2.18 |
| Detachment from others | .18 | .03 | .000 | 2.10 | .22 | 0.07 | .001 | 2.06 |
| Inability to experience positive emotions | .22 | .03 | .000 | 2.11 | .10 | 0.08 | .190 | 2.23 |
| Irritability | .05 | .03 | .066 | 1.48 | .29 | 0.07 | .000 | 2.00 |
| Self-destructive or reckless behavior | -.11 | .03 | .001 | 1.36 | .14 | 0.07 | .028 | 1.36 |
| Hypervigilance | .02 | .02 | .393 | 1.58 | .15 | 0.07 | .027 | 2.21 |
| Exaggerated startle response | .01 | .02 | .830 | 1.54 | -.22 | 0.07 | .002 | 2.25 |
| Concentration problems | .23 | .02 | .000 | 1.80 | .26 | 0.06 | .000 | 1.82 |
| Sleep disturbance | .11 | .02 | .000 | 1.72 | .05 | 0.06 | .419 | 1.85 |
| *Note.* *b* = unstandardized regression coefficient; *s.e.* = standard error; *p* = *p*-value; *VIF* = variance inflation factor. | | | | | | | | |

| SM4a  Differences between relative contributions – Norwegian sample | | | | | | | | | | | | | | | | | | | | |
| --- | --- | --- | --- | --- | --- | --- | --- | --- | --- | --- | --- | --- | --- | --- | --- | --- | --- | --- | --- | --- |
|  | Mem | Drm | Fls | Psy | Phy | Avm | Avx | Amn | Blf | Blm | Neg | Anh | Det | Pos | Irr | Rsk | Hyp | Str | Cnc | Slp |
| Mem |  |  |  |  |  |  |  |  |  |  |  |  |  |  |  |  |  |  |  |  |
| Drm | * |  |  |  |  |  |  |  |  |  |  |  |  |  |  |  |  |  |  |  |
| Fls | * |  |  |  |  |  |  |  |  |  |  |  |  |  |  |  |  |  |  |  |
| Psy |  | * | * |  |  |  |  |  |  |  |  |  |  |  |  |  |  |  |  |  |
| Phy |  | * | * | * |  |  |  |  |  |  |  |  |  |  |  |  |  |  |  |  |
| Avm | * |  |  | * | * |  |  |  |  |  |  |  |  |  |  |  |  |  |  |  |
| Avx |  |  |  | * |  |  |  |  |  |  |  |  |  |  |  |  |  |  |  |  |
| Amn | * |  | * | * | * | * | * |  |  |  |  |  |  |  |  |  |  |  |  |  |
| Blf | * | * | * | * | * | * | * | * |  |  |  |  |  |  |  |  |  |  |  |  |
| Blm |  | * | * |  |  | * |  | * | * |  |  |  |  |  |  |  |  |  |  |  |
| Neg | * | * | * | * | * | * | * | * | * | * |  |  |  |  |  |  |  |  |  |  |
| Anh | * | * | * | * | * | * | * | * | * | * |  |  |  |  |  |  |  |  |  |  |
| Det | * | * | * | * | * | * | * | * | * | * |  |  |  |  |  |  |  |  |  |  |
| Pos | * | * | * | * | * | * | * | * |  | * |  | * |  |  |  |  |  |  |  |  |
| Irr |  |  |  | * |  |  |  | * | * |  | * | * | * | * |  |  |  |  |  |  |
| Rsk | * | * | * | * | * | * | * |  | * | * | * | * | * | * | * |  |  |  |  |  |
| Hyp | * |  |  | * | * |  |  | * | * | * | * | * | * | * |  | * |  |  |  |  |
| Str | * |  |  | * | * |  |  |  | * | * | * | * | * | * |  | * |  |  |  |  |
| Cnc | * | * | * | * | * | * | * | * |  | * |  |  |  |  | * | * | * | * |  |  |
| Slp | * | * | * |  | * | * | * | * | * | * | * |  | * | * | * | * | * | * | * |  |
| *Note.* Asterisk indicates that 95% CI for difference did not include 0; mem = intrusive thoughts or memories, drm = distressing dreams, fls = dissociative flashbacks, psy = psychological cue reactivity, phy = physiological cue reactivity, avm = avoidance of thoughts or memories, avx = avoidance of external reminders, amn = trauma-related amnesia, blf = negative beliefs, blm = distorted blaming of oneself or others, neg = persistent negative emotional state, anh = diminished interest in activities, det = detachment from others, pos = inability to experience positive emotions, irr = irritability, rsk = self-destructive or reckless behavior, hyp = hypervigilance, str = exaggerated startle response, cnc = concentration problems, and slp = sleep disturbance. | | | | | | | | | | | | | | | | | | | | |

| SM4b  Differences between relative contributions – US sample | | | | | | | | | | | | | | | | | | | | |
| --- | --- | --- | --- | --- | --- | --- | --- | --- | --- | --- | --- | --- | --- | --- | --- | --- | --- | --- | --- | --- |
|  | Mem | Drm | Fls | Psy | Phy | Avm | Avx | Amn | Blf | Blm | Neg | Anh | Det | Pos | Irr | Rsk | Hyp | Str | Cnc | Slp |
| Mem |  |  |  |  |  |  |  |  |  |  |  |  |  |  |  |  |  |  |  |  |
| Drm |  |  |  |  |  |  |  |  |  |  |  |  |  |  |  |  |  |  |  |  |
| Fls |  |  |  |  |  |  |  |  |  |  |  |  |  |  |  |  |  |  |  |  |
| Psy |  |  |  |  |  |  |  |  |  |  |  |  |  |  |  |  |  |  |  |  |
| Phy |  |  |  |  |  |  |  |  |  |  |  |  |  |  |  |  |  |  |  |  |
| Avm |  |  |  |  |  |  |  |  |  |  |  |  |  |  |  |  |  |  |  |  |
| Avx |  |  |  |  |  |  |  |  |  |  |  |  |  |  |  |  |  |  |  |  |
| Amn |  |  |  |  |  | * |  |  |  |  |  |  |  |  |  |  |  |  |  |  |
| Blf | * | * | * | * | * | * | * |  |  |  |  |  |  |  |  |  |  |  |  |  |
| Blm |  |  |  |  |  |  |  |  | * |  |  |  |  |  |  |  |  |  |  |  |
| Neg |  | * |  | * |  | * |  |  | * | * |  |  |  |  |  |  |  |  |  |  |
| Anh | * | * | * | * | * | * | * | * |  | * | * |  |  |  |  |  |  |  |  |  |
| Det | * | * | * | * | * | * | * | * |  | * | * |  |  |  |  |  |  |  |  |  |
| Pos | * | * | * | * | * | * | * |  |  | * | * |  |  |  |  |  |  |  |  |  |
| Irr | * | * | * | * | * | * | * | * |  | * | * |  |  |  |  |  |  |  |  |  |
| Rsk |  | * |  | * |  | * |  |  |  | * |  | * | * |  | * |  |  |  |  |  |
| Hyp |  |  |  |  |  | * |  |  | * |  |  | * | * | * | * |  |  |  |  |  |
| Str |  |  |  |  |  |  |  |  | * |  | * | * | * | * | * | * |  |  |  |  |
| Cnc | * | * | * | * | * | * | * |  |  | * | * |  |  |  |  |  | * | * |  |  |
| Slp |  | * |  |  |  | * |  |  | * |  |  | * | * | * | * |  |  |  | * |  |
| *Note.* Asterisk indicates that 95% CI for difference did not include 0; mem = intrusive thoughts or memories, drm = distressing dreams, fls = dissociative flashbacks, psy = psychological cue reactivity, phy = physiological cue reactivity, avm = avoidance of thoughts or memories, avx = avoidance of external reminders, amn = trauma-related amnesia, blf = negative beliefs, blm = distorted blaming of oneself or others, neg = persistent negative emotional state, anh = diminished interest in activities, det = detachment from others, pos = inability to experience positive emotions, irr = irritability, rsk = self-destructive or reckless behavior, hyp = hypervigilance, str = exaggerated startle response, cnc = concentration problems, and slp = sleep disturbance. | | | | | | | | | | | | | | | | | | | | |

| SM5  Independent *t*-tests comparing (exclude cases analysis by analysis) comparing PTSS symptom severity between the 540 Norwegian participants with missings on one or more PTSS items (range: 1-19) and 3804 cases with no missings. | | | | | | | |
| --- | --- | --- | --- | --- | --- | --- | --- |
| DSM-5 posttraumatic stress symptoms | *Groups* | *n* | *M* | *SD* | *df* | *t* | *p* |
| Intrusive thoughts or memories | No PTSS missings  PTSS Missings | 3804  503 | 1.10  .97 | .98  .97 | 4305 | 2.813 | .005 |
| Distressing dreams | No PTSS missings  PTSS Missings | 3804  508 | .70  .58 | .93  .91 | 4310 | 2.551 | .011 |
| Dissociative flashbacks | No PTSS missings  PTSS Missings | 3804  470 | .64  .52 | .88  .84 | 4272 | 2.887 | .004 |
| Psychological cue reactivity | No PTSS missings  PTSS Missings | 3804  472 | 1.36  1.19 | 1.09  1.10 | 4274 | 3.140 | .002 |
| Physiological cue reactivity | No PTSS missings  PTSS Missings | 3804  468 | 1.09  .96 | 1.09  1.08 | 4270 | 2.563 | .010 |
| Avoidance of thoughts or memories | No PTSS missings  PTSS Missings | 3804  449 | 1.56  1.42 | 1.17  1.17 | 4251 | 2.402 | .016 |
| Avoidance of external reminders | No PTSS missings  PTSS Missings | 3804  420 | 1.20  1.04 | 1.18  1.17 | 4222 | 2.628 | .009 |
| Trauma-related amnesia | No PTSS missings  PTSS Missings | 3804  411 | .80  .80 | 1.03  1.04 | 4213 | .041 | .967 |
| Negative beliefs | No PTSS missings  PTSS Missings | 3804  421 | 1.30  1.22 | 1.18  1.16 | 4223 | 1.311 | .190 |
| Distorted blaming of oneself or others | No PTSS missings  PTSS Missings | 3804  412 | .91  .72 | 1.10  1.07 | 4214 | 3.276 | .001 |
| Persistent negative emotional state | No PTSS missings  PTSS Missings | 3804  416 | 1.34  1.18 | 1.12  1.14 | 4218 | 2.811 | .005 |
| Diminished interest in activities | No PTSS missings  PTSS Missings | 3804  385 | .94  .87 | 1.10  1.10 | 4187 | 1.173 | .241 |
| Detachment from others | No PTSS missings  PTSS Missings | 3804  356 | 1.00  .90 | 1.12  1.06 | 4158 | 1.653 | .098 |
| Inability to experience positive emotions | No PTSS missings  PTSS Missings | 3804  416 | .92  .89 | .99  1.01 | 4218 | .556 | .578 |
| Irritability | No PTSS missings  PTSS Missings | 3804  409 | .87  .90 | .98  .99 | 4211 | -.581 | .561 |
| Self-destructive or reckless behavior | No PTSS missings  PTSS Missings | 3804  382 | .39  .33 | .74  .71 | 4184 | 1.479 | .139 |
| Hypervigilance | No PTSS missings  PTSS Missings | 3804  387 | 1.04  .96 | 1.13  1.13 | 4189 | 1.342 | .180 |
| Exaggerated startle response | No PTSS missings  PTSS Missings | 3804  391 | .95  .95 | 1.11  1.12 | 4193 | .000 | 1.000 |
| Concentration problems | No PTSS missings  PTSS Missings | 3804  394 | 1.57  1.57 | 1.18  1.21 | 4196 | -.148 | .882 |
| Sleep disturbance | No PTSS missings  PTSS Missings | 3804  397 | 1.34  1.43 | 1.20  1.20 | 4199 | -1.343 | .179 |
| *Note. n* = sample size, *M* = mean, *SD* = standard deviation, *df* = degrees of freedom, *t* = *t*-test statistic, *p* = *p* value. | | | | | | | |
